# Performance and Mechanism of Co and Mn Loaded on Fe-Metal-Organic Framework Catalysts with Different Morphologies for Simultaneous Degradation of Acetone and NO by Photothermal Coupling

**DOI:** 10.3390/toxics12070524

**Published:** 2024-07-21

**Authors:** Yuanzhen Li, Yinming Fan, Yanhong Wang, Yinian Zhu, Zongqiang Zhu, Shengpeng Mo, Xiaobin Zhou, Yanping Zhang

**Affiliations:** 1 The Guangxi Key Laboratory of Theory and Technology for Environmental Pollution Control, Guilin University of Technology, Guilin 541000, China; 17771948725@163.com (Y.L.); 2428293972@163.com (Y.W.); zhuyinian@glut.edu.cn (Y.Z.); zhuzongqiang@glut.edu.cn (Z.Z.); moshengpeng14@mails.ucas.ac.cn (S.M.); zhouxiaobin@glut.edu.cn (X.Z.); 2 Collaborative Innovation Center for Water Pollution Control and Water Safety in Karst Area, Guilin University of Technology, Guilin 541004, China; 3 Guangxi Engineering Research Center of Comprehensive Treatment for Agricultural Non-Point Source Pollution, Guilin University of Technology, Guilin 541000, China; 4 Modern Industry College of Ecology and Environmental Protection, Guilin University of Technology, Guilin 541000, China

**Keywords:** volatile organic compounds, NO_x_, photothermal coupling, metal–organic skeleton, reaction mechanism

## Abstract

VOCs can be used instead of ammonia as a reducing agent to remove NO, achieving the effect of removing VOCs and NO simultaneously. Due to the high energy consumption and low photocatalytic efficiency required for conventional thermocatalytic purification, photothermal coupled catalytic purification can integrate the advantages of photocatalysis and thermocatalysis in order to achieve the effect of pollutants being treated efficiently with a low energy consumption. In this study, samples loaded with Co and Mn catalysts were prepared using the hydrothermal method on Fe-MOF with various morphologies. The catalytic performance of each catalyst was analyzed by studying the effects of their physicochemical properties through various characterizations, including XRD, SEM, BET, XPS, H_2_-TPR, TEM and O_2_-TPD. The characterization results demonstrated that the specific surface area, pore volume, high valence Co and Mn atoms, surface adsorbed oxygen and the abundance of oxygen lattice defects in the catalysts were the most critical factors affecting the performance of the catalysts. Based on the results of the performance tests, the catalysts prepared with an octahedral-shaped Fe-MOF loaded with Co and Mn showed a better performance than those loaded with Co and Mn on a rod-shaped Fe-MOF. The conversions of acetone and NO reached 50% and 64%, respectively, at 240 °C. The results showed that the catalysts were capable of removing acetone and NO at the same time. Compared with the pure Fe-MOF without Co and Mn, the loaded catalysts showed a significantly higher ability to remove acetone and NO simultaneously under the combination of various factors. The key reaction steps for the catalytic conversion of acetone and NO on the catalyst surface were investigated according to the Mars–van Krevelen (MvK) mechanism, and a possible mechanism was proposed. This study presents a new idea for the simultaneous removal of acetone and NO_x_ by photothermal coupling.

## 1. Introduction

Volatile organic compounds (VOCs) and nitrogen oxides (NO_x_) are atmospheric pollutants that should not be ignored. VOCs are released into the atmosphere through powerful diffusion force and chemical reaction with atmospheric nitrogen oxides, which will produce a large amount of photochemical smog and generate highly toxic gasses, thus causing secondary pollution, seriously impairing the quality of the atmospheric environment and threatening the safety of human life [1,2]. The synergistic control of pollutants such as VOCs and NO_x_ can effectively reduce pollutant emissions, thus promoting the continuous improvement of ambient air quality.

Currently, the related technologies, mainly photocatalysis and thermal catalysis, are the primary treatment methods for VOCs and NO_x_ pollutants [3]. The primary method of controlling VOCs is through catalytic degradation technology, whereas the management of NO_x_ is mainly based on the selective catalytic reduction (SCR) technology using ammonia as the reductant, but all of them suffer from the drawbacks of the narrow activity temperature window and the susceptibility to the secondary pollution of ammonia [4,5,6]. According to research [7], VOCs can be used instead of ammonia as a reductant for NO removal to realize the simultaneous removal of VOCs and NO. Due to the high energy consumption and low photocatalytic efficiency required for conventional thermocatalytic purification, photothermal coupled catalytic purification can integrate the advantages of photocatalysis and thermocatalysis to achieve the effect of efficiently treating pollutants with low energy consumption, and has advantages that conventional single photocatalysis or thermocatalysis do not have, which can make the removal rate of the target species significantly higher, and at the same time, it can improve the selectivity of the target products and reduce the temperature required for the reaction to overcome the disadvantages of each method while making use of the generated VOCs as a reductant. Their respective shortcomings while using the resulting synergistic effect to further improve the catalytic performance prevent the formation of efficient catalytic reactions [8,9,10]. Therefore, the development of greener, economical, low-temperature and efficient catalysts for the simultaneous degradation of VOCs and NO_x_ by photothermal coupling is promising and of great significance for solving the environmental pollution problem, especially when combined with the current research progress and practical application requirements.

Manganese oxide catalysts have attracted much attention due to their excellent oxygen storage capacity and redox properties [11]. Representatively, cobalt-modified manganese catalysts were investigated for the removal of nitric oxide, and the synergistic catalytic effect between manganese and cobalt was found to be the main reason for the high activity of the catalysts [12,13,14]. Meanwhile, MOF materials have been widely developed and applied in the field of catalyst preparation. Since the discovery of MOFs in the 1990s, MOF materials have attracted great interest and attention from scholars all over the world by virtue of their ultra-high specific surface area, controllable microscopic morphology and pore structure, topological diversity, abundant unsaturated coordination centers and the modifiability of their surfaces, especially in the fields of adsorption and catalysis [15,16,17,18]. Currently, researchers are gradually shifting their focus from MOFs to their derivatives because of the limitation of the low thermal stability of MOFs [19]. MOFs and their composites are gradually transformed into metal oxides by high-temperature heat treatment. The transformed MOF-based derivatives not only inherit the porosity and good morphology of MOF but also have better thermal stability, dispersion and dimensional homogeneity [20,21,22,23]. Their applications have been expanded, and they have been successfully applied in many fields, such as adsorption, separation, catalysis, sensing and so on [24,25,26,27,28]. They would also be good candidates as catalysts for the simultaneous degradation of VOCs and NO by photothermal coupling.

In this study, catalyst samples with different morphologies were prepared by Co and Mn loaded onto different morphologies of an Fe-MOF. Acetone, as a typical VOC, was used to replace ammonia as the reductant for the NO catalytic reduction, and the simultaneous removal of acetone and NO was achieved under photothermal conditions to avoid the secondary pollution of ammonia. Meanwhile, the physicochemical properties of the catalysts were explored by various analytical techniques, including XRD, BET, SEM, TEM, XPS, H_2_-TPR and O_2_-TPD. Based on the results of the performance tests, the catalysts prepared with an octahedral-shaped Fe-MOF loaded with Co and Mn showed a better performance than those loaded with Co and Mn on a rod-shaped Fe-MOF. The conversions of acetone and NO reached 50% and 64%, respectively, at 240 °C. The results showed that the catalysts were capable of removing acetone and NO at the same time. Compared with the pure Fe-MOF without Co and Mn, the loaded catalysts showed a significantly higher ability to remove acetone and NO simultaneously under the combination of various factors. Furthermore, the possible conformational relationship between the morphology and catalytic performance of the catalysts was determined. In addition, a possible reaction mechanism for the simultaneous removal of acetone and nitric oxide by the catalyst under photothermal co-catalysis was proposed based on the characterization data.

## 2. Materials and Methods

### 2.1. Preparation of Catalysts

FeCl_3_⋅6H_2_O, Mn(NO_3_)_2_ (50 wt%), Co(NO_3_)_2_⋅6H_2_O, and ransbutylene dioic acid were purchased from Shanghai Aladdin Biochemical Science and Technology Co. (Shanghai, China), Ltd. 2,5-dihydroxyterephthalic acid (H4dhtp) and N, N-dimethylformamide (DMF) were purchased from Shanghai Een Chemical Technology Co. (Shanghai, China). Ethanol (EtOH) was purchased from Tianjin Fuyu Fine Chemical Co. (Tianjin, China). All the above reagents were of analytical grade and did not require further purification. Ultrapure water was produced by our laboratory.

#### 2.1.1. Preparation of Carriers

(1) Preparation of octahedral shaped Fe-MOF

The samples were prepared using the hydrothermal method. First, 3.75 mmol FeCl_3_·6H_2_O (1.01 g) and 1.875 mmol H_2_BDC (terephthalic acid) (0.31 g) were added to 45 mL of DMF (N, N-dimethylformamide) and mixed in order to prepare the precursor solution, and the reaction mixture was magnetically stirred for 30 min at room temperature. The mixed solution was then transferred to a 100 mL PTFE-lined stainless steel autoclave, and the reaction temperature was set at 110 °C and the reaction time was set to 24 h. At the end of the reaction, after the autoclave had cooled down to room temperature, the precipitate was collected by filtration, washed with deionized water three times and finally dried in a constant temperature oven at 80 °C for 12 h. The reaction was carried out at a constant temperature of 80 °C.

(2) Preparation of Fe-MOF in stick form

We dissolved 10 mmol FeCl_3_⋅6H_2_O (2.70 g) and 10 mmol transbutylene dioic acid (1.16 g) in 100 mL of deionized water to form a clarified solution. Then, the clarified solution dissolved in FeCl_3_⋅6H_2_O was poured into the solution dissolved in transbutylene dioic acid, and then stirred vigorously for 30 min at room temperature. Finally, the mixture solution was transferred to a round-bottomed flask and kept in an oil bath at 100 °C for 4 h. The reaction was carried out in a circular-bottomed flask. When the reaction mixture had naturally cooled to room temperature, the precipitate was collected by filtration, washed with ethanol 3 times, then washed with deionized water 3 times and finally dried in a constant temperature drying oven at 80 °C for 12 h.

#### 2.1.2. Preparation of Catalysts with Different Carriers Loaded with Co and Mn

We dissolved 0.13 g C_8_H_6_O_6_ (2, 5-dihydroxyterephthalic acid) in 60 mL of DMF–ethanol (C_2_H_5_OH)–water mixture (DMF (53 mL), C_2_H_5_OH (3.5 mL) and H_2_O (3.5 mL)) with a volume ratio of 15:1:1 (*v*/*v*/*v*), and then 430 μL Mn(NO_3_)_2_ and 0.11 g Co(NO_3_)_2_⋅6H_2_O (n_Co:Mn_ = 2:1) were added to the mixed solution and stirred for 10 min at room temperature; then, 0.2 g of the prepared Fe-MOF (octahedral) and Fe-MOF (stick) were used as templates, respectively, and dispersed into the mixed solution, magnetically stirred for 10 min and ultrasonicated for 20 min. The mixed solution was transferred to a 100 mL flask. The reaction temperature was set at 135 °C, and the reaction time was 24 h. After the autoclave had cooled down to room temperature, the precipitate was collected by filtration, washed with ethanol 3 times, then with deionized water 2 times and dried in a constant temperature oven at 80 °C for 12 h. Finally, the precursor was put into a quartz tube, and the tube furnace was heated to a temperature of 2 °C/min at an elevated rate of 2 °C/min to a temperature of 1 °C/min with a heating rate of 500 °C for 2 h. The precursors were named sample A and sample B, respectively.

### 2.2. Catalyst Characterization

The prepared catalysts were analyzed by various characterization methods including X-ray powder diffraction (XRD, Shanghai, China), Brunauer–Emmett–Teller (BET, Beijing, China), scanning electron microscopy (SEM, Zeiss, Sigma300, Wuxi, China), energy dispersive X-ray attachment (EDS, Oxford Xplore50, Guangzhou, China), high-resolution transmission electron microscopy (HRTEM-SAED, JEOLJEM-2100F, Wuxi, China), Transmission electron microscopy (TEM, JEOLJEM-2100F, Wuxi, China), X-ray photoelectron spectroscopy (XPS, Axis Ultra DLD, Kratos, Shanghai, China), H_2_-TPR, O_2_-TPD and UV–visible diffuse reflection spectra (UV–Vis DRS, Wuhan, China). Please refer to the Supplementary Materials for more information.

### 2.3. Catalytic Evaluation

We weighed 300 mg (40–60 mesh) of catalyst and placed it in a quartz tube filled with cotton, and then the quartz tube was mounted into an atmospheric fixed-bed continuous-flow reactor under UV irradiation (280 nm < λ < 380 nm) and vented with a gas mixture containing acetone (5 ppm) and NO (500 ppb) (20 vol% O_2_/Ar), with a total gas flow rate of 1000 mL/min. The gas volumetric space velocity (GHSV) was 120,000 mL·g^−1^·h^−1^. The reaction temperature was controlled by the programmed temperature increase method, and the temperature points for data recording were taken as 80 °C, 110 °C, 140 °C, 170 °C, 200 °C, 210 °C, 220 °C, 230 °C and 240 °C, and the temperature increase rate was 5 °C/min, and the holding time for each temperature point was 20. The holding time of each temperature point was 20 min.

The concentrations of pre-reaction and post-reaction gasses were tested by an online GC-6600 gas chromatograph (Fanwei, Shanghai, China) and a low-concentration automatic smoke analyzer (Junray, model ZR-3260D, Qingdao, China). The catalytic performance of each catalyst was reacted by calculating the conversion of acetone and *NO* as follows:

(1) $$\eta_{CH_{3}COCH_{3}}=\frac{C_{CH_{3}COCH_{3},in}-C_{CH_{3}COCH_{3},out}}{C_{CH_{3}COCH_{3},in}}\times 100\%$$

(2) $$\eta_{NO}=\frac{C_{NO,in}-C_{NO,out}}{C_{NO,in}}\times 100\%$$

In the formula, $\eta_{CH_{3}COCH_{3}}$, $C_{CH_{3}COCH_{3},in}$, $C_{CH_{3}COCH_{3},out}$, $\eta_{NO}$, $C_{NO,in}$ and $C_{NO,out}$ are used to express the conversion rate of acetone, the initial concentration of acetone (ppm), the outlet concentration of acetone (ppm), the conversion rate of *NO*, the initial concentration of *NO* (ppb) and the outlet concentration of *NO* (ppb), respectively.

## 3. Results and Discussion

### 3.1. Structural Characterization of the Catalyst

**Figure 1:**
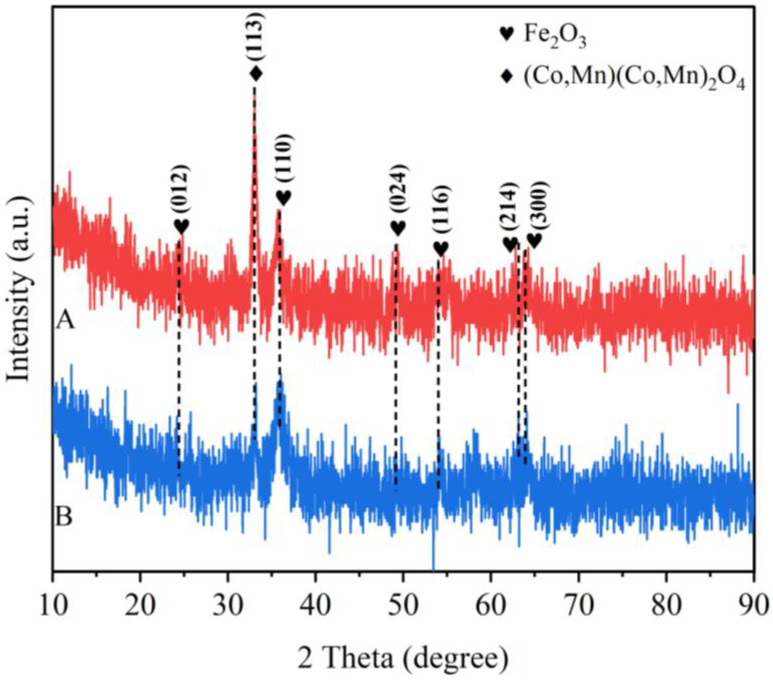
XRD spectra of each catalyst.

The crystalline structure of the catalysts was characterized by XRD, and the exposed crystal faces and crystal clusters of the catalysts were analyzed, as shown in Figure 1. The analysis of the spectra shows that there is one characteristic peak at 33.0° for both Sample A and Sample B, which corresponds to the (113) crystalline facet of (Co, Mn) (Co, Mn)_2_O_4_ (JCPDS PDF#18-0408) [29,30,31], and there are five characteristic peaks at 24.2°, 35.9°, 49.1°, 54.1° and 64.1°, which correspond to the crystal facets of Fe_2_O_3_ (JCPDS PDF#85-0599) in the (012), (110), (024), (116) and (300) crystal planes; in addition, sample A also has one more characteristic peak at 62.8° than sample B, which corresponds to the (214) crystal plane of Fe_2_O_3_ (JCPDS PDF#85-0599) [32,33,34]. Sample B has a similar peak position to sample A, but the peak corresponding to the crystal face is relatively high and narrow. Based on the XRD spectra of sample A, it can be seen that its crystallinity is lower than that of sample B, which favors the formation of more exposed planes and more lattice defects.

**Figure 2:**
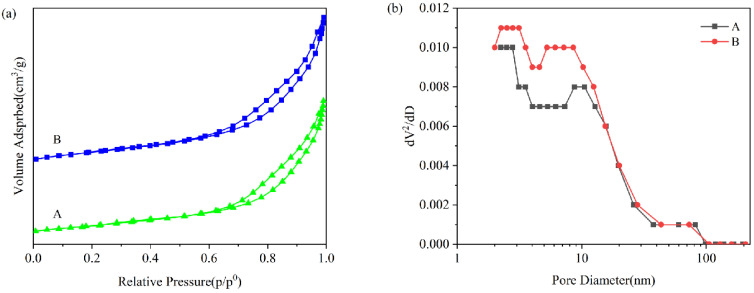
N_2_ adsorption–desorption isotherms (**a**) and pore size property distributions (**b**) for each catalyst.

The specific surface areas and pore structures of catalysts are some of the influencing factors that affect the catalytic performance of catalysts [35]. The specific surface area and pore structure of each catalyst were tested using BET, and the relevant data are presented in Table 1. Figure 2a shows the N_2_ adsorption–desorption isotherms of each catalyst. It was obvious that the adsorption–desorption isotherms of all catalysts were typical type IV isotherms with H3-type hysteresis loops, indicating the presence of mesoporous structures in these samples. The non-coincidence of adsorption and desorption curves of isotherms at higher relative pressures is caused by the capillary condensation phenomenon [36,37]. Figure 2b shows the BJH adsorption pore volume–pore size distribution of each catalyst. Combined with Table 1, it can be seen that these samples have similar pore volume and pore size distributions, and the pore size is mainly concentrated in the mesopore range of 2–50 nm. The kinetic diameter of acetone is 0.45 nm, which belongs to the larger VOC molecules, and the mesopore size of the pores is favorable for the reaction [38]. As shown in Table 1, the specific surface area and pore volume are A > B, and the average adsorption pore size is A < B. The BET test results were different for Sample A compared with Sample B because both of them added different morphologies of Fe-MOF. Compared with sample B, sample A has a smaller particle size and more uniform dispersion, so the specific surface area of the catalyst is relatively high. The average adsorbed pore size of Sample A is relatively small (13.88 nm), which indicates that this sample has smaller pores and a poorer crystallinity. These properties promote the formation of lattice defects and increase the oxygen vacancy concentration [39]. Sample A has a larger BET-specific surface area (71.14 m^2^/g) and pore volume (0.24 m^2^/g), which is favorable for providing more activation sites during the catalyst reaction. For the catalyst, the larger specific surface area and pore volume enhanced the surface adsorption rate of the reactants. They provided a larger reaction space for the reaction process, which resulted in an improved VOC removal rate [40]. This is also in perfect agreement with the results of the subsequent photothermal catalysis performance tests.

**Figure 3:**
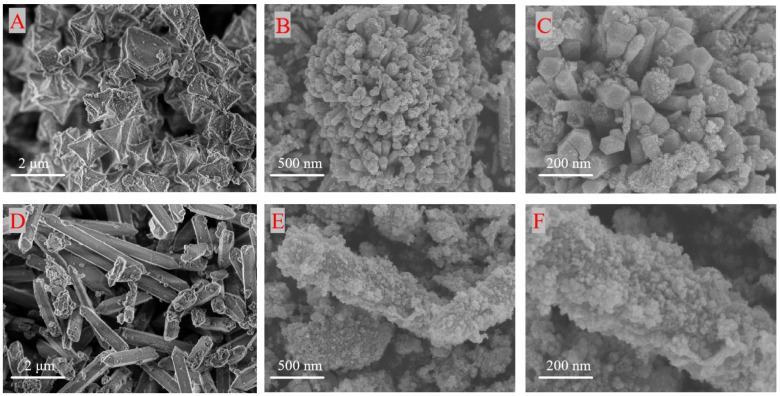
SEM images of Fe-MOF (octahedral shape) (**A**), Fe-MOF (rod shape) (**D**), Sample A (**B**,**C**), and Sample D (**E**,**F**).

**Figure 4:**
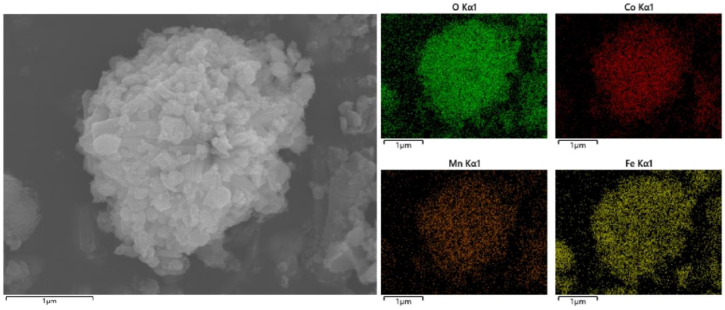
SEM-EDS plot of Sample A.

**Figure 5:**
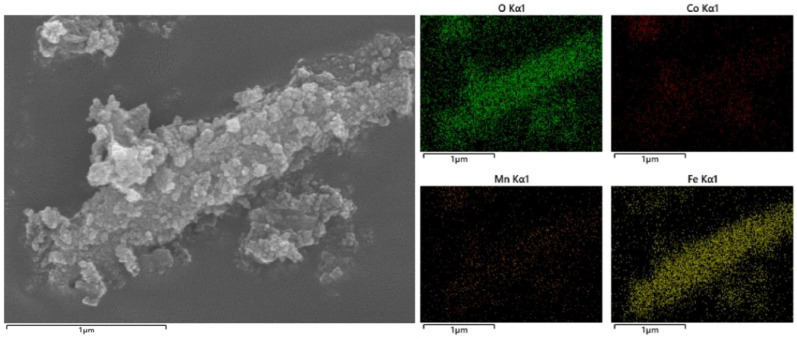
SEM-EDS image of Sample B.

The morphology of each catalyst was investigated by scanning electron microscopy (SEM), as shown in Figure 3: A is the SEM image of Fe-MOF (octahedral shape), BC is Sample A, D is the Fe-MOF (rod shape) and EF is Sample B. The SEM images of the catalysts are shown in Figure 3. It can be seen that Sample A has a homogeneous spherical morphology with microspheres consisting of aggregated rods, which is changed from the octahedral shape of Fe-MOF when it is not loaded with Co, Mn; Sample C has a long rod shape consisting of nanospheres, which is not changed from the rod shape of Fe-MOF when it is not loaded with Co, Mn. SEM-EDS maps of Samples A and B are shown in Figure 4 and Figure 5, respectively. The elemental mapping of the two further shows that the elemental distribution of the catalysts is relatively homogeneous, and the three-dimensional structure of each catalyst has a high thermal stability.

**Figure 6:**
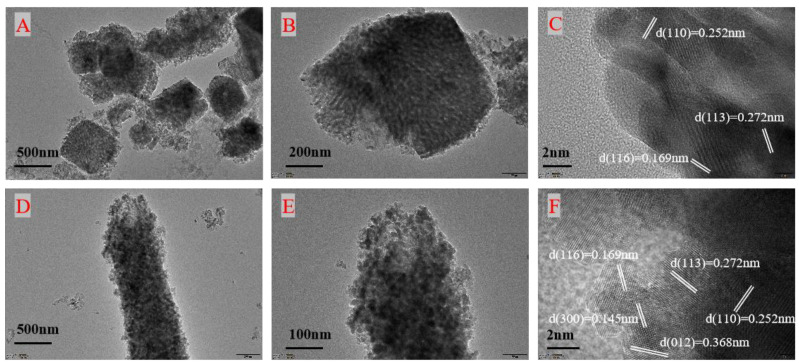
TEM images of Sample A (**A**–**C**) and Sample B (**D**–**F**).

TEM was used to further investigate the morphology and microstructure of each catalyst. Figure 6 shows the HRTEM images of Samples A and B (ABC, DEF), respectively. As can be seen from the figures, the calculated plane spacings of the lattice fringes of Sample A were 0.169 nm, 0.252 nm and 0.272 nm, which matched with the (116), (113) and (110) planes of the spinel phase, and the plane spacings of the lattice fringes of Sample B were 0.145 nm, 0.196 nm, 0.252 nm, 0.272 nm and 0.368 nm, matching the (300), (116), (110), (113) and (012) planes of the spinel phase [29,34,35]. The HRTEM test results are in good agreement with the SEM and XRD results.

**Figure 7:**
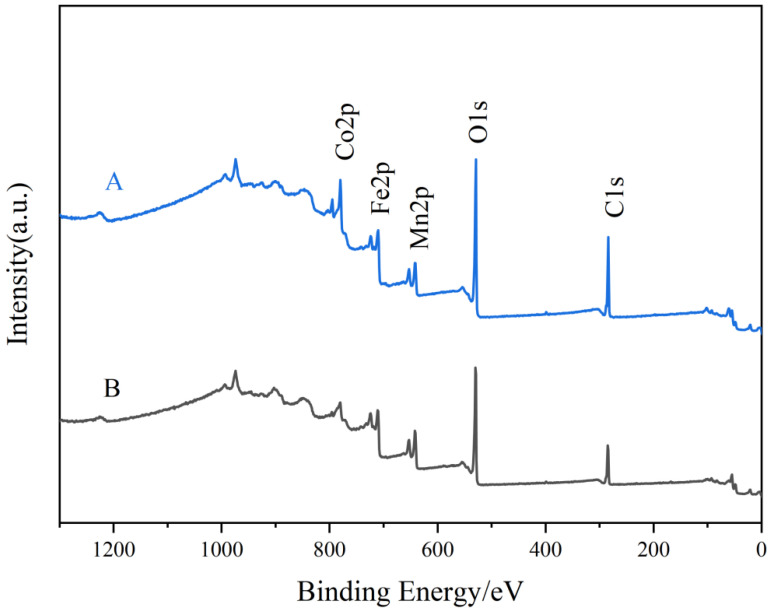
Full spectrum of catalyst.

**Figure 8:**
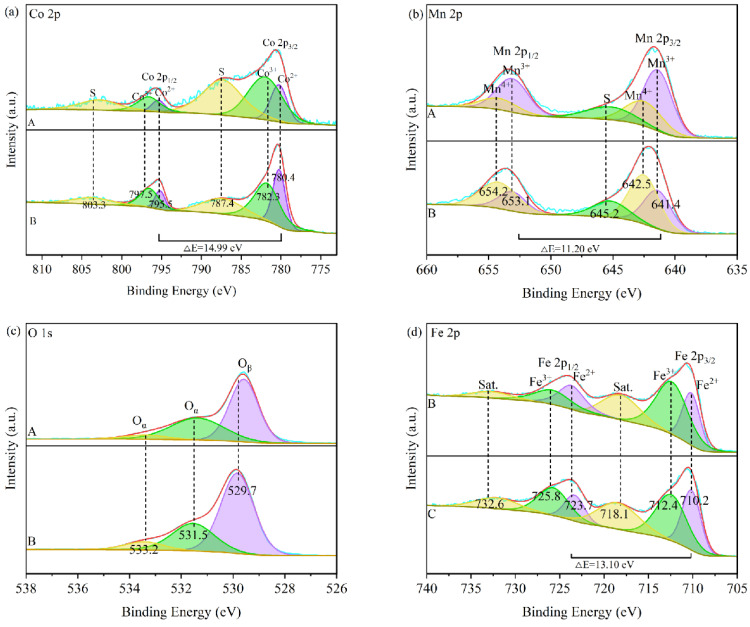
XPS accurate spectra of each element of the catalyst: (**a**) Co 2p; (**b**) Mn 2p; (**c**) O 1s; (**d**) Fe 2p.

XPS characterization was used to analyze the surface elemental composition of the catalysts and the valence distribution of each element. Figure 7 shows the full spectrum of each catalyst. Figure 8 shows the XPS fine spectra of Co 2p, Mn 2p, O 1s and Fe 2p. The figure shows that Mn, Co, C, O and Fe are the main elements in the compositions of Samples A and B.

As shown in Figure 8a, Co 2p is a spin-orbit double state consisting of Co 2p1/2 and Co 2p3/2, with a binding energy difference between them of about 15.1 eV. The Co 2p1/2 spectrum of each catalyst consists of two peaks, Co^2+^ (796.9 eV) and Co^3+^ (795.3 eV), and the Co 2p3/2 spectra consist of two peaks, Co^2+^ (781.4 eV) and Co^3+^ (780.0 eV) peaks [41]. According to previous studies, cobalt (Co^3+^) in the high valence state participates in the oxidation reaction of VOCs as an active component, so Co^3+^ plays an essential role in the oxidative degradation of acetone [42,43].

As shown in Figure 8b, Mn 2p is a spin-orbit double state consisting of Mn 2p1/2 and Mn 2p3/2, with a binding energy difference of about 11.7 eV between them, and the Mn 2p1/2 spectra of each catalyst consisted of two peaks of Mn^4+^ (653.0 eV) and Mn^3+^ (651.2 eV), and the Mn 2p3/2 spectra consisted of two peaks of Mn^4+^ (642.3 eV) and Mn^3+^ (640.9 eV) peaks. The failure to fit the Mn^2+^ peaks for Samples A and B may be due to the presence of MnO_x_, Co_3_O_4_ and FeO_x_ interacting with each other through electron transfer. Similar to Co^3+^, the high valence state of manganese (Mn^4+^) favors the oxidation reaction of VOCs, and Mn^4+^ plays an important role in the oxidation reaction of VOCs [41,44,45].

Figure 8c shows the fine O 1s spectra of each catalyst. The O 1s spectra can be deconvoluted into three peaks corresponding to different types of oxygen on the surface of each catalyst. Among them, the peak located at 529.8 eV belongs to the lattice oxygen species (O_β_) related to the peak located at 531.6 eV and 533.4 eV belongs to the surface-adsorbed oxygen (O_α_) related to the peak [46]. Generally, the surface-adsorbed oxygen plays a vital role in the catalytic oxidation system because it can directly participate in the oxidation reaction in the system and promote the oxidation reaction, thus further accelerating the total oxidation of VOCs, which is of significant positive significance for the oxidation reaction [47].

Figure 8d shows the fine spectra of Fe 2p for each catalyst. Fe 2p is a spin-orbit double state consisting of Fe 2p1/2 and Fe 2p3/2, and the difference in binding energy between the two is about 13.1 eV. The Fe 2p1/2 spectra of each catalyst consisted of two peaks of Fe^2+^ (723.7 eV) and Fe^3+^ (725.8 eV), and the Fe 2p3/2 spectra consisted of two peaks of Fe^2+^ (710.2 eV) and Fe^3+^ (712.4 eV). There is also a higher BE shoulder peak near 718.4 eV, which may be related to the interaction between Fe^2+^ and Fe^3+^. It has been shown that Fe^2+^ has a positive effect on the co-processing of VOCs and NO_x_ [48].

In conclusion, combined with Table 1, it can be seen that, for Mn^4+^/Mn^3+^, Co^3+^/Co^2+^, O_α_/(O_α_ + O_β_) (%) and Fe^2+^/Fe^3+^, A > B. Sample A has a smaller percentage of Fe^2+^/Fe^3+^ compared with Sample B. At the same time, both of them are mixed-valent systems consisting of a Co system and a Mn system and the high-valent atoms of the two elements can be known to play an important role in the photothermal synergistic catalytic system. It can be seen that the high valence atoms of the two elements play an important role in this photothermal co-catalytic system, and the high ratio of Co^3+^ and Mn^4+^ atoms is conducive to the oxidation reaction of this system. This is the main reason that the catalytic performance of Sample A is better than that of Sample B, and both of them have a better redox performance, a higher number of oxygenation vacancy defects and a more significant activity than their Fe-MOF catalyst counterparts without the introduction of Co and Mn [49,50,51].

**Figure 9:**
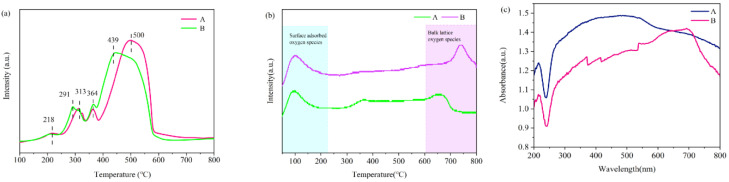
H_2_-TPR spectra (**a**), O_2_-TPD spectra (**b**) and UV-vis spectra (**c**) for each catalyst.

The redox properties of each catalyst were analyzed by H_2_-TPR and O_2_-TPD characterization. Figure 9a shows the H_2_-TPR plots of each catalyst, from which it can be seen that each catalyst exhibits two main reduction zones (200~350 °C, 350~600 °C), in which the reduction zone at 200~350 °C is mainly contributed by the reduction of MnO_2_ or Mn_2_O_3_ to Mn_3_O_4_ as well as the surface Co_3_O_4_ to CoO and the reduction of Fe_2_O_3_ to FeO, while the reduction zone at 350~600 °C is mainly contributed by the reduction of non-surface Co_3_O_4_ to CoO and the reduction of CoO to Co^0^ as well as the reduction of Mn_3_O_4_ to MnO_2_, and by the reduction of FeO to Fe^0^. It is clear from the observations that the peaks in the range of 100–200 °C are related to the reduction of surface oxygen species. The shaded area indicates that the peak area of Sample A is the largest, which suggests that more oxygen vacancies are formed on Sample A, which further promotes the catalytic reaction and, therefore, leads to a better low-temperature reduction capability [52,53]. In addition, the trend in the reduction capacity of each catalyst in the reduction zone coincides with the trend in the performance of each catalyst in photothermal-catalyzed acetone and NO conversion.

The O_2_-TPD can characterize the composition of oxygen species on the catalyst surface. Figure 9b shows the O_2_-TPD plots for each catalyst, from which it can be seen that each catalyst exhibited three obvious oxygen desorption peaks in the temperature intervals of 0~200 °C, 200~600 °C and 600~800 °C, respectively. The oxygen desorption peaks in the temperature range of 0~200 °C were attributed to the release of physically adsorbed oxygen and physicochemically adsorbed oxygen weakly bound to the catalyst surface; the oxygen desorption peaks in the temperature range of 200~600 °C were attributed to the surface lattice oxygen generated by the fracture of unsaturated Co-O, unsaturated Mn-O and Fe-O. The oxygen desorption peaks in the temperature range of 600~800 °C were attributed to the bulk oxygen generated from the valence transition of cobalt, manganese and iron oxides under the high-temperature treatment. The oxygen desorption peaks in the 600~800 °C temperature region are attributed to the lattice oxygen in the bulk phase generated by the valence transition of cobalt, manganese, and iron oxides at high temperatures [32,34,41]. Previous research has shown that physically adsorbed oxygen and physicochemically adsorbed oxygen weakly bound to the catalyst surface play an important role in enhancing the oxidation capacity of catalysts, and oxygen lattice defects play a non-negligible role in enhancing the photothermal conversion capacity and reducibility of the catalysts [54,55].

In general, within a photothermal catalytic system, the stronger the light absorption capacity of a catalyst is, the more favorable the photothermal catalytic performance of that catalyst is [56,57]. Figure 9c shows the UV-vis spectra of each catalyst, from which it can be seen that the catalysts have similar absorption spectra in the UV and visible wavelength ranges but with different light absorption capacities. The light absorption ability of Sample A is better than that of Sample B. By comparing with the photothermal catalytic performance of the catalyst, it is found that the stronger the light absorption ability of the catalyst in this system, the better the photothermal catalytic performance. This indicates that photocatalysis is a crucial factor in the conversion of NO and acetone in this system. From this, the photothermal synergistic mode of this system is photo-assisted thermo-catalysis (PATC), i.e., heat is the main driving force of the reaction, and light assists in promoting the thermo-catalytic process [58].

### 3.2. Analysis of the Photothermal Catalytic Activity of Catalysts

**Figure 10:**
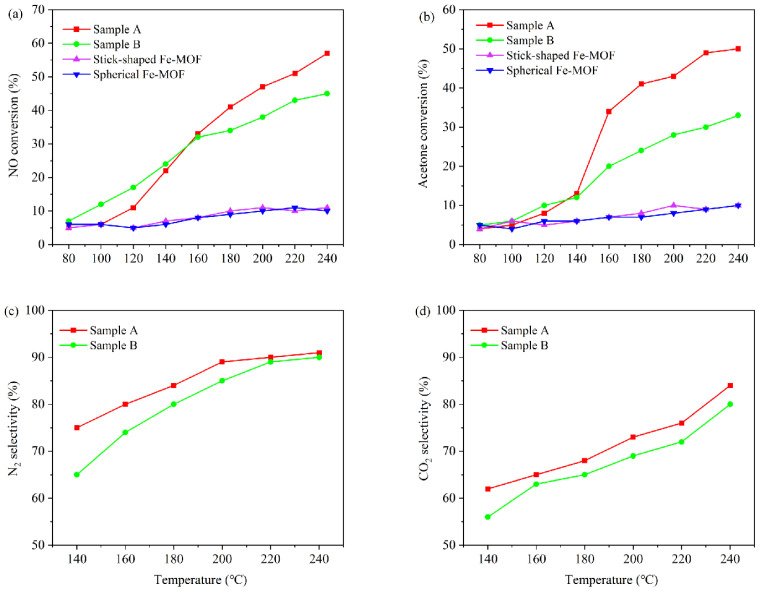
NO conversion (**a**), acetone conversion (**b**), N_2_ selectivity (**c**) and CO_2_ selectivity (**d**) for each catalyst.

All the catalysts prepared above were applied in a photothermal co-catalytic reaction system under UV irradiation to investigate the photothermal catalytic performance of the samples obtained by different preparation methods. In this system, NO was used as the oxidant and acetone as the reductant. The prepared samples A and B were used as the catalysts. The simultaneous conversion of NO and acetone was achieved without additional NH_3_ flux, and the removal rates of NO and acetone are shown in Figure 10a,b, respectively. Meanwhile, the conversion rates of NO and acetone by Fe-MOFs with different morphologies unloaded with Co and Mn are also reflected in Figure 10a,b. From the figure, it can be seen that the removal of NO and acetone by the two catalysts without loading of Co and Mn is not much different, which is around 10%, and the performance of the catalysts is poor. However, the catalytic performance of the loaded catalyst was significantly improved by the introduction of Co and Mn elements. It can be assumed that the simultaneous presence of Co and Mn can produce a synergistic effect, thus improving the performance of the catalyst. This indicates that Co_3_O_4_ and MnO_x_ are the key factors that allow this type of catalyst to have a catalytic conversion ability. The used catalyst still had a relatively high activity, suggesting the structure of the catalyst was not changed after activity evaluation (Figure S1). The conversion of NO and acetone by sample B was slightly higher than that of sample A at 80–140 °C. Also, in conjunction with Figure 9b,c, it can be argued this may be due to the fact that the light absorption ability of Sample B and its own oxygen lattice defects are superior to those of A at this stage, which is the main reason affecting the photothermal catalytic performance of the catalysts at this stage. With the increase in temperature, the conversion of NO and acetone by Sample A gradually increased, which was higher than that of Sample B. Sample A had a higher percentage of Co^3+^, Mn^4+^ and Fe^2+^ atoms and a higher content of O_2_ ads compared to Sample B, which was favorable for the oxidation reaction in this system. Hence, the performance of this catalyst was better than that of Sample B as a whole [53].

### 3.3. Analysis of Photothermal Catalysis Mechanism of Catalysts

Based on the above study, a possible mechanism for the simultaneous conversion of acetone and *NO* in this system was proposed. In this system, heat is the main driving force for the catalytic oxidation reaction, and the *MnO*_2_, *Co*_3_*O*_4_ and *Fe*_2_*O*_3_ in the catalyst are heated to obtain thermal energy directly. In addition, the reaction temperature of the system can be further increased by absorbing light, which in turn promotes the activation of surface-adsorbed oxygen *O*_2_ ads and surface lattice oxygen *O* lattices. Surface lattice oxygen is the main oxidation site in the system and is an important oxidant in the catalytic oxidation reaction, while surface cobalt, surface manganese and surface iron may be the main adsorption sites in the system, and the catalytic oxidation of acetone on the surface of the catalysts follows the Mars–van Krevelen (MvK) mechanism [41,44,45,54].

(3) $$NO+e^{-}\to NO^{-}$$

(4) $$O_{2}(ads)+e^{-}\to •O_{2}^{-}(ads)$$

(5) $$O_{2}(ads)+2H_{2}O(ads)\to 4•OH(ads)$$

(6) $$CH_{3}COCH_{3}+MnO_{2}\to CH_{3}COO(ads)+HCOO(ads)+MnO_{2-x}+H_{2}O$$

(7) $$CH_{3}COCH_{3}+Co_{3}O_{4}\to CH_{3}COO(ads)+HCOO(ads)+Co_{3}O_{4-y}+H_{2}O$$

(8) $$CH_{3}COCH_{3}+Fe_{2}O_{3}\to CH_{3}COO(ads)+HCOO(ads)+Fe_{2}O_{3-z}+H_{2}O$$

(9) $$CH_{3}COO(ads)+•O_{2}^{-}(ads)\to CO^{-}+H_{2}O$$

(10) $$HCOO(ads)+•OH(ads)\to CO_{2}+H_{2}O$$

(11) $$CO^{-}+h^{+}\to CO$$

(12) $$CO+2NO^{-}+2h^{+}\to CO_{2}+N_{2}$$

(13) $$MnO_{2-x}+O_{2}(ads)\to MnO_{2}$$

(14) $$Co_{3}O_{4-y}+O_{2}(ads)\to Co_{3}O_{4}$$

(15) $$Fe_{2}O_{3-z}+O_{2}(ads)\to Fe_{2}O_{3}$$

Briefly, as the temperature in the system increases and light is applied, the active components of the catalyst are excited with electron (*e*^−^) and hole (*h*^+^) pairs. *NO* and *O*_2_ (*ads*) capture the free *e*^−^ to form the more reactive *NO*^−^ and ·*O*^2−^ (*ads*), and the *e*^−^,·*h*^+^ pairs react with *O*_2_ (*ads*) adsorbed on the surface of the catalyst and *H*_2_*O* (*ads*) to form ·*OH*(*ads*). According to the Mars–van Krevelen (MvK) mechanism, acetone adsorbed on the catalyst surface reacts with the surface lattice oxygen and is rapidly converted to *CH*_3_*COO*(*ads*) and *HCOO*(*ads*), while *MnO*_2_, *Co*_3_*O*_4_, and *Fe*_2_*O*_3_ are combined with oxygen lattice defects to form *MnO*_2−*x*_, *Co*_3_*O*_4−*x*_, and *Fe*_2_*O*_3−*x*_. This step is the tachycritical step in the catalytic reaction. The tachycritical step of the whole catalytic reaction and determines whether the catalytic oxidation reaction can be carried out efficiently. Subsequently, *CH*_3_*COO*(*ads*) reacts with ·*O*^2−^(*ads*) on the surface of the catalyst to form *CO*^−^ and *H*_2_*O*, and *HCOO*(*ads*) reacts with ·*OH*(*ads*) on the surface of the catalyst to form *CO*_2_ and *H*_2_*O*. the generated *CO*^−^ rapidly transfers *e*^−^ to the hole, and then reacts with *NO*^−^ to form *CO*_2_ and *N*_2_. The oxygen lattice defects of *MnO*_2−*x*_, *Co*_3_O_4−*x*_ and *Fe*_2_O_3−*x*_ are the key factors in the catalytic oxidation reaction. *Co*_3_*O*_4−*x*_ and *Fe*_2_*O*_3−*x*_ will react with oxygen adsorbed on the catalyst surface to generate *MnO*_2_, *Co*_3_*O*_4_ and *Fe*_2_*O*_3_.

## 4. Conclusions

In summary, we have synthesized oxide catalysts with different morphologies derived from Fe-MOFs and catalysts loaded with Co and Mn as their carriers by hydrothermal method and compared them with bit-loaded Fe-MOF for comparative study, and each catalyst was used for the simultaneous removal of acetone and NO under the low-temperature photothermal synergistic catalytic system in which acetone was substituted for NH_3_. The performance test results showed that the catalysts loaded with Co and Mn in an octahedral-shaped Fe-MOF performed better than those loaded with a rod-shaped Fe-MOF. The catalyst achieved high conversion rates for acetone and NO of up to 50% and 64%, respectively, at 240 °C with UV irradiation. Compared with the pure Fe-MOF without Co or Mn loading, the loaded catalyst has a significantly higher capacity for the simultaneous removal of acetone and NO under the combined effect of multiple factors. It is illustrated that the microstructure, surface structure, structural defects, redox properties and optical properties of each catalyst were analyzed through a series of characterizations, and it was found that the photothermal catalytic system was light-assisted thermo-catalytic and that heat was the main driving force for the catalytic oxidation reaction. The main factors affecting the performance of the catalyst are the adsorbed oxygen on the surfaces of Co, Mn and Fe in the high valence states of the catalyst and the abundance of oxygen lattice defects, and the catalyst’s specific surface area, light-absorption capacity and the photogenerated carrier separation capacity also have a certain effect on the performance of the catalyst. Finally, a possible mechanism for the catalytic conversion of acetone and NO on the catalyst surface was proposed based on the characterization results and the Mars–van Krevelen (MvK) mechanism. During the decomposition of acetone, the small molecule intermediates methane, ethane and acetaldehyde acted as reducing agents in our reaction system. This study provides a new idea for obtaining a photothermal catalyst for efficient simultaneous removal of acetone and NO at a low cost.

## Figures and Tables

**Table 1:** BET data for each catalyst and atomic percentage and elemental valence content of Co 2p, Mn 2p, O 1s, Fe 2p in each catalyst.

Catalyst	S_BET_ (m^2^·g^−1^)	V_pore_ (cm^3^·g^−1^)	Pore Diameter (nm)	Mn^4+^/Mn^3+^	Co^3+^/Co^2+^	Fe^3+^/Fe^2+^	O_α_/(O_α_ + O_β_) (%)
A	71.14	0.24	13.88	1.43	1.41	0.65	43.5%
B	59.15	0.22	15.51	0.92	1.04	0.51	36.0%

## Data Availability

The datasets used or analyzed during the current study are available from the corresponding authors upon reasonable request.

## Supplementary Materials

The following supporting information can be downloaded at: https://www.mdpi.com/article/10.3390/toxics12070524/s1, Figure S1: Catalytic performance for the used materials.
