# Supplementary material for: Performance and Mechanism of Co and Mn Loaded on Fe-Metal-Organic Framework Catalysts with Different Morphologies for Simultaneous Degradation of Acetone and NO by Photothermal Coupling
Source: Toxics. 2024 Jul 21;12(7):524. doi: 10.3390/toxics12070524 (PMC11281022; doi:10.3390/toxics12070524)
Supplement: Supplementary file 1 [file toxics-12-00524-s001.zip › toxics-3089429-supplementary.pdf]

1.Characterization

The X-ray powder diffraction (XRD) were conducted on a D8 instrument (BrukerAXS) with Cu Kα radiation (40 kV, 40 mA, scanning step = 0.02).

The N_2 adsorption-desorption isotherms of the samples were measured via N_2 adsorption at -196 °C on an automatic surface analyzer (Micromeritics ASAP 2020). Automatic rapid specific surface area and mesopore / micropore analyzer (BET): to determine the specific surface area, pore volume, pore size of catalyst samples. In the automatic surface analyzer (JW-BK200C), heat and vacuum the sample (200°C, 4 h), and then determine the N_2 adsorption-desorption isotherm of the sample.

The surface morphology of the catalysts were measured by the scanning electron microscopy (SEM, Zeiss, Sigma300) with an energy dispersive X-ray attachment (EDS, Oxford Xplore50).

The microscopic morphology and crystal structure of the catalysts were analyzed by the high-resolution transmission electron microscopy (HRTEM-SAED, JEOLJEM-2100F).

Transmission electron microscopy (TEM): the microtissue structure of the sample at an accelerating voltage of 200 kV.

X-ray photoelectron spectroscopy (XPS, Axis Ultra DLD, Kratos) were acquired at a basic pressure 3.33 × 10^{-6} Torr with Mg Kα radiation (hv = 1253.6 eV) at 15 kV. The binding energy calibration was performed using the C 1 s peak in the background as the reference energy (284.6 eV).

For H_2-TPR, 50 mg catalyst was first treated under an Ar flow (30mL min^{-1}) at 300 °C for 1 h to remove physically adsorbed molecules, and then cooled down to room temperature. Subsequently, the gas was switched to a flow of 10 vol% H_2/Ar (30 mL min^{-1}) in the tube and the sample was heated to 800 °C at a ramp of 10 °C min^{-1}.

For O_2-TPD, 50 mg catalyst was first treated under an He flow (30 mL min^{-1}) at 300 °C for 1 h to remove physically adsorbed molecules, and then cooled to room temperature. Then, the gas was switched to a flow of 5 vol% O_2/He (30 mL min^{-1}) in the tube for 1 h, followed by purging with pure He at the same temperature for 30 min to remove the unabsorbed O_2 molecules. Then, the catalysts were heated from room

temperature to 750 °C at 10 °C min^{-1} in pure He (30 mL min^{-1}).

For UV-vis, the emission intensity was measured while the excitation wavelength steps up with an increment of 10 nm for each scan. The slit widths for both excitation and emission were set at 5 nm. The fluorescence excitation scans were performed using the same instrument.

2. Supplementary figures

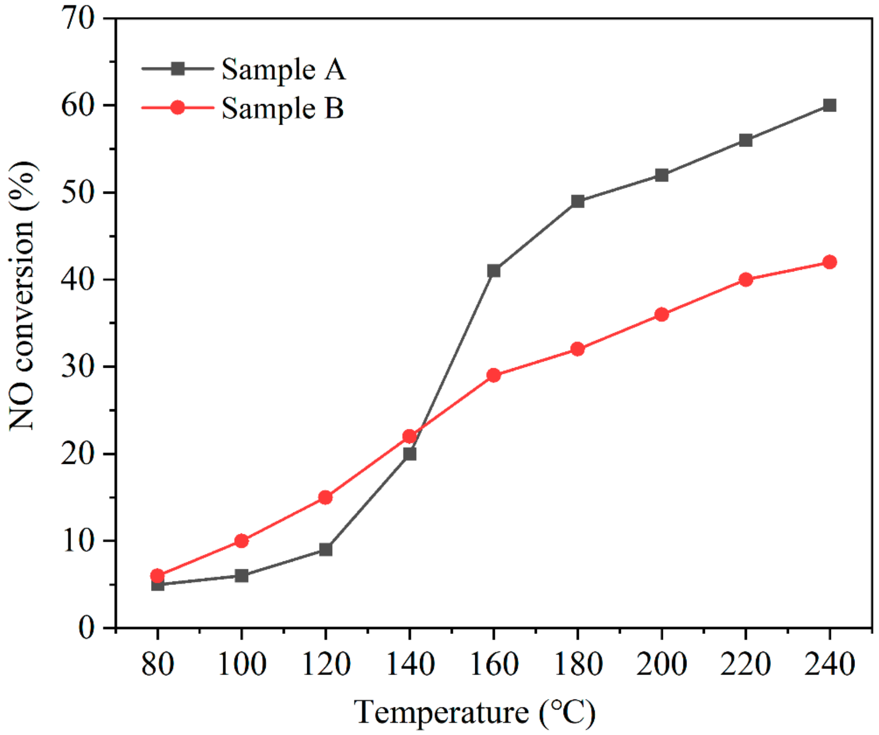

Figure S1. Catalytic performance for the used materials
